# Beyond weight loss: digital therapeutic for behavioral change and psychological well-being for individuals with overweight and obesity in a primary healthcare setting—A randomized controlled pilot study

**DOI:** 10.3389/fdgth.2025.1671649

**Published:** 2025-09-16

**Authors:** Marthe Isaksen Aukan, Maria Arlèn Larsen, Tone Iren Melan, Øyvind Olav Salvesen

**Affiliations:** ^1^ Obesity Research Group, Department of Clinical and Molecular Medicine, Faculty of Medicine, Norwegian University of Science and Technology (NTNU), Trondheim, Norway; ^2^ Exercise, Cardiometabolic Health and Reproduction (EXCAR) Research Group, Department of Circulation and Medical Imaging, Norwegian University of Science and Technology (NTNU), Trondheim, Norway; ^3^ Department of Clinical Medicine, UiT The Arctic University of Norway, Tromsø, Norway; ^4^ Healthy Lifes Center, Stjørdal Municipally, Stjørdal, Norway; ^5^ Department of Public Health and Nursing, Faculty of Medicine, Norwegian University of Science and Technology (NTNU), Trondheim, Norway

**Keywords:** obesity, healthy lifestyle, behavioral change, mental health, mHealth, DTx

## Abstract

**Objective:**

Mobile health (mHealth) through digital therapeutics (DTx) offer a promising approach to obesity management. This study evaluated the effectiveness of the Lifeness DTx for obesity care and its effect on anthropometrics, reward-related eating behaviors and quality of life in individuals with overweight and obesity within a community-based healthcare setting.

**Methods:**

A 12-week randomized controlled trial was conducted. Adults (BMI ≥ 27 kg/m^2^, and central obesity) were recruited from municipal Healthy Life Centers in Norway. The intervention group (IG) received standard care plus full DTx app with program functionality and digital follow-up, whereas the control group (CG) received standard care with limited app functions and no DTx program. Outcome variables were measured at baseline and after 12 weeks.

**Results:**

No significant changes in body weight, or differences between groups were observed at W12. The IG showed reductions in waist circumference (−3.4 cm, *p* = 0.008, *d* = −0.926), waist-to-height ratio (−0.02, *p* = 0.008, *d* = −0.929), improvements on hedonic eating behavior, indicated by reduced disinhibition (−1.6, *p* = 0.013, *d* = −0.907), as well as increased quality of life (+5.0, *p* = 0.019, *d* = 0.899). Both groups increased self-esteem (IG +9.8, *p* = 0.018, *d* = 0.911, and CG +12, *p* = 0.050, *d* = 0.838).

**Conclusion:**

The DTx intervention was associated with improvements in central adiposity, reward-related eating behaviors, and psychological well-being beyond weight loss. These findings provide preliminary evidence that digital therapeutics may represent a feasible and scalable approach to support personalized obesity care in primary healthcare settings. Larger, adequately powered trials are needed to confirm these results.

**Clinical Trial Registration:**

clinicaltrials.gov, identifier NCT06667843 (Initial Release: 10/15/2024).

## Introduction

Obesity is a chronic, progressive, and relapsing disease (1) defined by excessive fat accumulation, or a body mass index (BMI) ≥ 30 kg/m^2^ (2). Obesity is stigmatized, associated with reduced health-related quality of life, depression and anxiety, is an independent risk factor for type 2 diabetes and cardiovascular diseases (3), and increases the risk of 31 types of cancers (4). Altogether contributing to obesity being one of the most expensive diseases (5). Despite this, obesity is underrecognized and sub-optimally addressed compared to other non-communicable diseases (6).

Clinically meaningful weight loss can be achieved by many with lifestyle interventions (7), but long-term weight management represents the greatest challenge (8, 9). A proposed “Behavioral Balance Model” (10) highlights the necessity of multimodal therapeutic approaches in obesity management. While top-down cognitive control mechanisms, such as dietary restraint and inhibitory control, can be improved through lifestyle interventions, they are often insufficient to counteract the bottom-up drive to eat (10). Combined with the limited effectiveness of dietary and physical activity advice (7) and insufficient support from healthcare professionals (11), this highlights how challenging sustainable lifestyle adaptions can be in our obesogenic environment.

Healthy Lives Centers in Norway are a part of the primary healthcare in municipalities. These centers are established to offer low threshold services that help citizens become more physically active, improve their diet, smoking cessation, improve sleep and mental health. But financial constraints restrict the availability of such services, including geographical availability (12). In addition, healthcare providers may also lack sufficient obesity care training, nutritional knowledge, and the latest insights on therapy developments (13).

Recent findings indicate that both pragmatic implementation of an automated online behavioral obesity treatment program with active maintenance phase (14), and a non-dietary psychological app program focusing on satiety perception (15) led to significant weight loss at 12- and 24 months, respectively. Furthermore, the role of nutritional management is evolving to embrace a more holistic and personalized approach (16). So rather than solely emphasizing weight loss, these shifts prioritize long-term, patient-centered strategies that recognize the multifaceted nature of obesity (17). And as such, Healthy Lives Centers in Norway exhibit a unique position to incorporate innovative and scalable interventions to effectively prevent and manage obesity and its comorbidities in a community-based setting.

This study represents the first to investigate the efficacy and scalability potential of a digital therapeutic (DTx) strategy for obesity in a primary healthcare setting in Norway. The primary objective was to address the complexity of obesity using a DTx with nutrition-, physical activity-, and behavioral therapy, including digital follow-up by Healthy Lives Centers healthcare professionals.

## Materials and methods

### Study design

The “Smart Nutrition, Healthier Communities” study is a two-arm randomized controlled pilot trial assessing the feasibility and effectiveness of digital therapeutic intervention as add-on to standard care in four Healthy Lives Centers in the region of Værnes in central Norway. Participants were randomized (1:1) by block randomization with stratification by BMI categories (27.0–34.9 kg/m^2^, and ≥35 kg/m^2^), using eFORSK, a web-based system developed and administered by Helse Midt-Norge information technology (IT) (Central Norway Regional Health Authority's IT department). Recruitment and data collection took place between September 2024 and January 2025. The study was approved by the regional ethics committee (Regionale komiteer for medisinsk og helsefaglig forskningsetikk—REK-midt, ref: 774938), registered at Clinicaltrials.gov 10/15/2024 (NCT06667843), and conducted according to the guidelines laid down in the Declaration of Helsinki. All participants provided written informed consent before enrolling in the study. This paper reports changes in body weight, central adiposity, reward-related eating behavior traits and quality of life. An outline of the study can be seen in Supplementary Figure S1 in Supplementary Materials.

### Participants

Adult men and women from the local community were recruited through the Healthy Lives Centers. Participants were screened for eligibility criteria before enrollment in the study: Aged 18 or older, BMI ≥27 kg/m^2^, and central obesity measured by waist circumference (≥88 cm for women, and ≥102 cm for men) (18), current weight stability (±2.5 kg self-reported weight change during the past three months), motivated to lifestyle change using mobile apps and access to smartphone with 5G. Exclusion criteria included: previous bariatric surgery, use of anti-obesity drugs, pregnancy, current or present cancer diagnosis, substance abuse or psychiatric diagnosis (such as eating disorders), and other conditions that can hinder physical activity. Participants were randomized into (1) Intervention group: Standard care plus full program DTx app functionality and digital follow-up, or (2) Control group: Standard care with basic (limited app functions).

### Standard care

**Figure 1:**
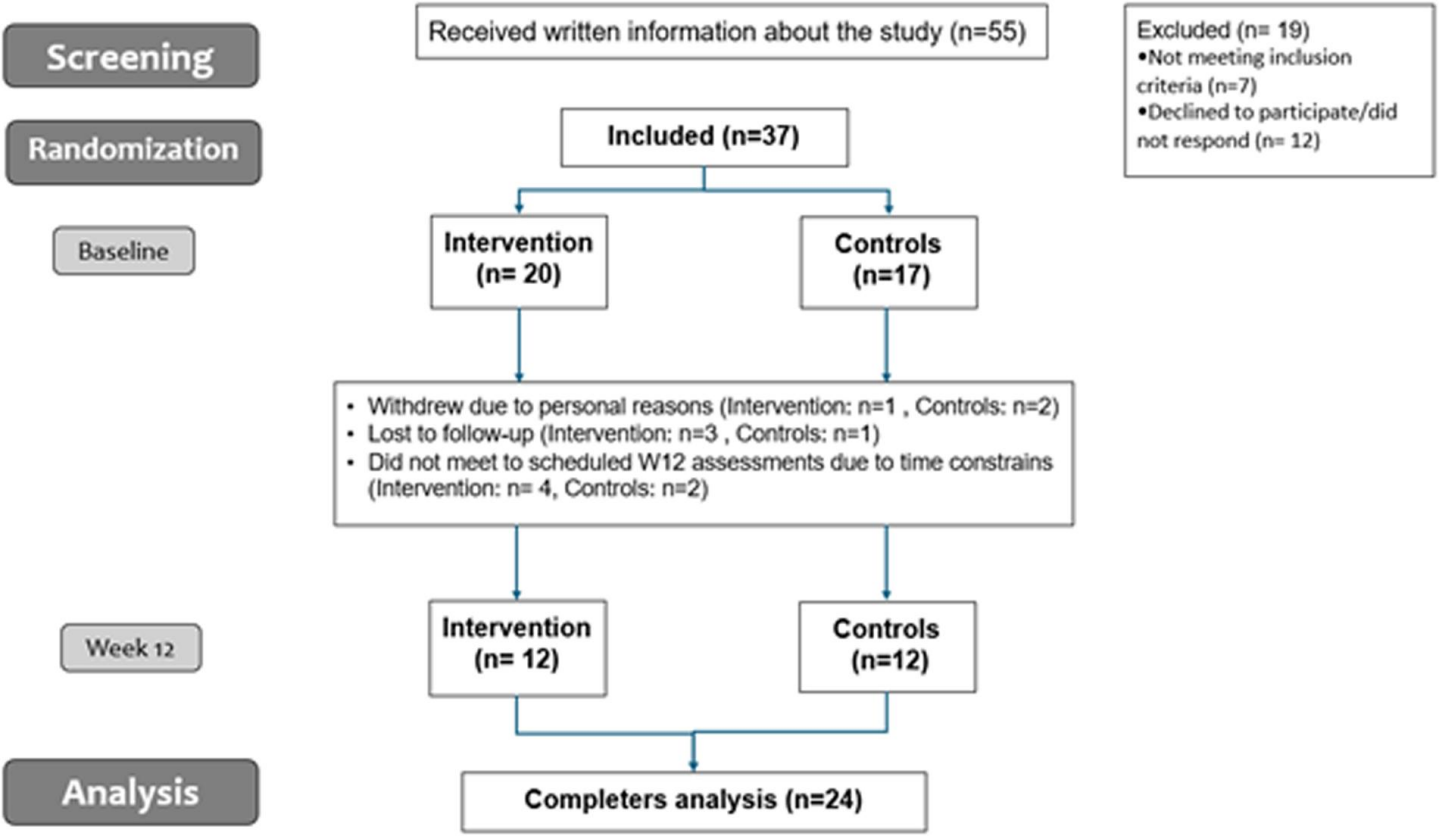
Flow chart of the study.

All participants attended a start-up meeting with their respective healthcare professionals at the Healthy Lives Centers as standard practice. Physical follow-up meetings could be scheduled as needed, and all participants were welcomed to attend group-based physical activity classes, and other lifestyle-related classes scheduled by the Healthy Lives Centers in the respective municipalities. A flowchart of the study can be seen in Figure 1.

### Digital therapeutic strategy

Lifeness is a mobile health (mHealth) platform for individuals with obesity that promotes self-management and patient compliance through personalized, evidence-based strategies. Lifeness can be used as an app alone, with- or without learning modules (DTx program). The app can be connected to a health care professional, as for this study: the Healthy Lives Centers. For this study, the program was adapted to fit the 12-week Healthy Lives Centers prescription in Norway, aimed at holistic lifestyle adaptions beyond weight loss for individuals with overweight or obesity. The program encouraged sustainable adaptions through regular self-monitoring and SMART goal setting (Specific, Measurable, Achievable, Relevant, Time-bound) (19), behavioral nudging to enhance autonomy and decision-making, and new knowledge modules every week. The modules included tasks such as screening forms to map individual strengths and barriers, articles, workout programs, and motivational videos covering topics such as mental health, sleep, nutrition, stress, and physical activity. Artificial Intelligence (AI) tools, such as food photo recognition diary and the possibility to connect smart devices, simplified logging and self-monitoring behaviors. The modules adapted to the patient's progress, with options for extending or unlocking new learning modules via gamification.

The intervention group (IG) had access to the full Healthy Lives Centers DTx package, and their healthcare providers were connected to the patients via a web-panel and could monitor their progress including safe communication as needed. The control group (CG) only had access to the logging functions in the app, and without digital follow up or-communication with the healthcare providers. All of the participants had access to a chatbot that could support them on different aspects of their behavioral change. Lifeness holds a CE-mark (MDR I), is ISO certified and approved by the Norwegian Directory of Health as a Safe Health App.

## Outcome variables

Participants came to their respective Healthy Lives Center, in the Værnes region at baseline (BL), and after 12 weeks (W12). Body weight was measured in kg with light clothing, using a digital medical scale (Soehnle S20, Soehnle Industrial Solutions GmbH), and waist circumference measured using standard procedures (rounding to the nearest 0.5 cm). All participants were asked to fill out the following questionnaires at BL and W12.

The Three Factor Eating Questionnaire (TFEQ) and Dutch Eating Behavior Questionnaire (DEBQ) was used to assess eating behavior traits. The TFEQ measures dietary restraint, disinhibition, and hunger (20). The DEBQ measures restrained-, external- and emotional eating (21). Emotional eating was divided into two sub-categories: diffuse emotions and clearly labelled emotions. The Impact of Weight on Quality of Life (IWQOL)-Lite questionnaire (22) was used to assess obesity-specific quality of life and includes 5 subcategories: physical function, self-esteem, sexual life, public distress and work. In addition, the total quality of life score was measured.

## Statistical analysis

The sample size was based on recommendations for pilot trials (23), aimed at testing feasibility, refining methods, and generating effect size estimates for future adequately powered trials. Statistical analyses were performed using SPSS, version 29 (SPSS Inc., Chicago, IL). Data is presented as estimated marginal means and -mean differences (W12-BL) with 95% confidence intervals (CI). Significance level was set to *P* < 0.05. No intention to treat analysis were performed. Data from completers were analyzed using a linear mixed-effects model with restricted maximum likelihood estimation, and a paired sample t-test to estimate within group effects, and effect sizes using Cohens d. Residuals were checked for normality using Shapiro Wilk test and by visual inspection of QQ plots and histograms.

## Results

### Participants

Table 1 shows mean characteristics of the participants at BL and W12. Thirty-seven participants completed BL assessments; twenty-four participants completed W12 assessments. Reasons for attrition rates are shown in Figure 1. Only completers were included in the analysis (*n* = 24). At BL, participants had an average age of 44 years, a BMI of 36.5 ± 1.0 kg/m^2^, and were mainly women (83%). Participants weighed on average 105.9 ± 4.1 kg, had a waist circumference of 110.6 ± 2.2 cm, and a waist/height ratio of 0.65 ± 0.0. No statistically significant differences were seen between groups for anthropometric variables at W12 (*p* > 0.05, for all) (Supplementary Table S1). For exploratory analysis, mean differences within group can be seen Table 2. No significant differences over time were observed for CG (*p* *>* *0.05, for all*). There was a trend for a modest reduction in body weight and BMI with moderate effect sizes from BL to W12 in the IG, but did not reach significance (*p* = 0.071, and *p* = 0.070 respectively). Waist circumference and waist/height ratio decreased in the IG (*p* = 0.008, for both) and with large effects sizes (Cohen's *d* = −0.926, and −0.929 respectively).

**Table 1:** Participant characteristics.

Characteristics	Baseline (all)	95%CI	Week 12 intervention	(95%CI)	Week 12 control	(95%CI)
*N*	24		12		12	
Age	43.9	(39.8, 48.1)				
Females (%)	83%		83%		83%	
Weight (kg)	105.9 ± 4.1	(97.5, 114.3)	104.5 ± 4.2	(95.9, 113.0)	104.3 ± 4.2	(95.7, 113.0)
BMI (kg/m^2^)	36.5 ± 1.0	(34.4, 38.5)	35.9 ± 1.0	(33.9, 38.1)	35.9 ± 1.0	(33.8, 37.9)
Waist (cm)	110.6 ± 2.2	(106.3, 115.0)	107.3 ± 2.3	(102.6, 112.0)	108.6 ± 2.3	(103.9, 113.3)
Waist/height ratio	0.65 ± 0.0	(0.63, 0.67)	0.63 ± 0.0	(0.60, 0.65)	0.64 ± 0.0	(0.62, 0.67)

Data presented as estimated marginal means ± standard error of the mean, and 95% confidence interval (CI).

Significance level <0.05.

BMI, body mass index; W12, week 12.

**Table 2:** Changes over time in anthropometric variables.

Anthropometrics	Group	Mean difference	SD	95% CI of the difference		*p*-value	Cohen’s d	95% CI	
				Lower	Upper			Lower	Upper
Weight (kg)	Intervention	−1.4	2.5	−3.0	0.1	*0*.*071*	−0.576	−1.179	0.049
	Control	−1.5	3.6	−3.8	0.8	0.170	−0.424	−1.007	0.177
BMI (kg/m^2^)	Intervention	−0.5	0.9	−1.1	0.1	*0*.*070*	−0.580	−1.183	0.045
	Control	−0.6	1.3	−1.4	0.4	0.161	−0.434	−1.019	0.168
Waist (cm)	Intervention	−3.4	3.6	−5.7	−1.1	**0**.**008**	−0.926	−1.595	−0.229
	Control	−1.8	3.8	−4.2	0.6	0.132	−0.470	−1.059	0,137
Waist to height ratio	Intervention	−0.02	0.02	−0.04	−0.10	**0**.**008**	−0.929	−1.598	−0.231
	Control	−0.01	0.02	−0.02	0.00	0.146	−0.451	−1.038	0.153

Data shown as mean differences (Week 12—Baseline) with 95% confidence interval (CI). Significance level set to <0.05. Bold indicates statistical significance (*p* ≤ 0.05). Effect sizes estimated with the standard deviation of the mean difference (Cohen’s d).

BMI, body mass index; SD, standard deviation.

### Eating behavior traits

No statistically significant differences between groups were seen at W12 on any eating behavior trait, results can be seen in Supplementary Table S2 in Supplementary Materials. For exploratory analysis Table 3 shows mean differences in TFEQ scores over time within group. No significant differences were observed for CG for the TFEQ. There was a trend towards an increase in dietary restraint for the intervention group (*p* = 0.096). Disinhibition significantly decreased for the intervention group (*p* = 0.013) with a large effect size (−0.907). No significant effects were seen for hunger. Table 4 shows mean differences in DEBQ scores within group over time. No significant effects were seen for any group for the DEBQ. CG showed a trend for reductions in emotional eating, restrained eating and for clearly labelled emotions (*p* = 0.075, *p* = 0.087, and *p* = 0.075, respectively) and with moderate effect sizes (−0.568, −0.543, −0.568, respectively). A trend for reduction in diffuse emotions (*p* = 0.053) with a moderate effect size (−0.661), was found IG.

**Table 3:** Changes in TFEQ scores over time.

TFEQ	Group	Mean difference	SD	95% CI of the difference		*p*-value	Cohen’s d	95% CI	
				Lower	Upper			Lower	Upper
Dietary restraint	Intervention	2.3	4.1	−0.5	5.0	0.096	0.554	−0.095	1.180
	Control	1.2	3.3	−0.9	3.3	0.250	0.351	−0.241	0.927
Disinhibition	Intervention	−1.6	1.8	−2.9	−0.4	**0**.**013**	−0.907	−1.601	0.182
	Control	−0.4	2.6	−2.1	1.2	0.586	−0.162	−0.728	0.411
Hunger	Intervention	−0.9	2.8	−2.0	1.8	0.918	−0.032	−0.622	0.560
	Control	−1.1	3.1	−3.0	0.9	0.245	−0.329	−0.931	0.238

Data shown as mean differences (Week 12—Baseline) with 95% confidence interval (CI). Significance level set to <0.05. Bold indicates statistical significance (*p* ≤ 0.05). Effect sizes estimated with the standard deviation of the mean difference (Cohen’s d).

SD, standard deviation; TFEQ, three factor eating behaviour questionnaire.

**Table 4:** Changes in DEBQ scores over time.

DEBQ	Group	Mean difference	SD	95% CI of the difference		*p*-value	Cohen’s d	95% CI	
				Lower	Upper			Lower	Upper
Emotional eating	Intervention	−0.2	0.5	−0.5	0.2	0.307	−0.325	−0.924	0.290
	Control	−0.2	0.3	−0.4	0.1	*0*.*075*	−0.568	−0.853	0.055
Restrained eating	Intervention	0.2	0.5	−0.2	0.5	0.266	0.355	−0.264	0.957
	Control	0.3	0.5	−0.1	0.6	*0*.*087*	0.543	−0.076	1.141
External eating	Intervention	−0.2	0.5	−0.5	0.1	0.121	−0.511	−1.130	0.131
	Control	−0.1	0.4	−0.4	0.2	0.512	−0.196	−0.763	0.380
Clearly labelled emotions	Intervention	−0.1	0.6	−0.5	0.3	0.646	−0.143	−0.734	0.455
	Control	−0.2	0.3	−0.4	0.1	*0*.*075*	−0.568	−1.170	0.055
Diffuse emotions	Intervention	−0.3	0.5	−0.7	−0.0	***0***.***053***	−0.661	−1.304	0.009
	Control	−0.1	0.6	−0.5	−0.2	0.354	−0.279	−0.851	0.304

Data shown as mean differences (Week 12—Baseline) with 95% CI. Significance level set to <0.05. Bold indicates statistical significance (*p* ≤ 0.05). Effect sizes estimated with the standard deviation of the mean difference (Cohen’s d).

DEBQ, dutch eating behaviour questionnaire; SD, standard deviation.

### Impact of weight on quality of life (IWQOL-lite)

No statistically significant differences between groups were observed at W12, results can be seen in Supplementary Table S3 in Supplementary Materials. For exploratory analysis Table 5 shows mean differences over time for IWQOL-lite scores, within group. Both IG and CG showed a significant improvement in self-esteem from BL to W12 (*p* = 0.018, and *p* = 0.050, respectively), and with large effect sizes (0.911, and 0.838, respectively). The IG also showed a significant improvement in the total score quality of life (*p* = 0.019) with a large effect size (0.899). No within group effects were seen on the other parameters of the IWQOL-lite.

**Table 5:** Changes in IWQOL-lite scores over time.

IWQOL-lite	Group	Mean difference	SD	95% CI of the difference		*p*-value	Cohen’s d	95% CI	
				Lower	Upper			Lower	Upper
Physical function	Intervention	3.0	8.8	−3.2	9.4	0.307	0.343	−0.305	0.973
	Control	4.3	7.1	−1.1	9.8	0.101	0.617	−0.279	0.889
Self-esteem	Intervention	9.8	10.8	2.1	17.5	**0**.**018**	0.911	0.148	1.639
	Control	12.0	14.4	0.0	24.1	**0**.**050**	0.838	0.001	1.633
Work	Intervention	1.2	13.5	−8.5	10.9	0.778	0.092	−0.532	0,711
	Control	0.1	11.9	−11.0	11.01	0.998	0.001	−0.740	0,742
Public	Intervention	−0.5	4.9	−4.1	3.1	0.758	−0.101	−0.719	0.524
	Control	3.5	9.9	−5.6	12.7	0.376	0.361	−0.419	1.115
Total score	Intervention	5.0	5.5	1.0	8.9	**0**.**019**	0.899	0.140	1.625
	Control	7.3	15.3	−4.4	19.1	0.187	0.480	−0.226	1.160

Data shown as mean differences (Week 12—Baseline) with 95% CI. Significance level set to <0.05. Bold indicates statistical significance (*p* ≤ 0.05). Effect sizes estimated with the standard deviation of the mean difference (Cohen’s d).

IWQOL-lite, impact of weight on quality of life—lite; SD, standard deviation.

## Discussion

This 12-week study is, to our knowledge, the first to suggest improvements in multiple behavioral aspects beyond weight loss for individuals with overweight or obesity, when adding a DTx solution to usual care in in a municipal Healthy Lives Center setting. While no differences were seen between groups in any variables, several within group effects were detected. The IG showed significant, but modest, reductions in central adiposity (waist circumference and waist-to-height ratio), and improvements in reward-related eating behaviors, quality of life and self-esteem.

The latest report from the Norwegian Directorate of Health reveal that the population's eating habits are far from national dietary recommendations, significantly contributing to the burden of disease (24). At the same time, the cost and health risks of obesity and overweight are shown to have substantial impacts on healthcare costs (25). A recent study also estimated that healthcare expenditures will increase significantly towards 2050, whereas stroke, diabetes, and cardiovascular diseases will account for a large share of this increase (26), and all closely related to dietary factors. Nutritional counseling by dietitians is shown to yield significant health benefits beyond weight loss (16), including improvements in metabolic and cardiovascular health, gut microbiome dysbiosis, inflammation, sleep quality, mental health, and overall quality of life. Equal access to obesity care, however, remains an important global issue (27), and impactful preventive measures on both an individual and societal level are lacking.

Monitoring and supporting the patient at all phases of the treatment cycle remain a valuable, effective and cost-effective tool for expanding access to obesity care for a larger patient population through modern technology (28–30). A growing body of evidence shows that mHealth lifestyle interventions using self-monitoring have positive effects on both anthropometric measures and behavioral components when compared to usual care in the short-term (31). Although, no significant between group effects were seen post-intervention in the present study, several statistically significant within group changes signaling clinical relevance were observed for the IG.

In the present study, participants showed a fairly modest, but non-significant reduction in body weight and BMI (for both groups) during the 12-week intervention. Although ≥5% weight loss is seen as clinically relevant, a recently published systematic review has shown that also smaller reductions in body weight is clinically meaningful for this population (32). Notably, the IG experienced reductions in waist circumference. While a decrease of 3.4 cm may seem modest, reductions in waist circumference are generally considered a proxy for reduced central adiposity and cardiometabolic risk (33). It also needs to be emphasized that the DTx did not guide participants in any group towards any caloric target for weight loss. And notably, the IG was nudged towards healthy food choices and -eating habits through the DTx program and a balanced macronutrient distribution according to national dietary guidelines (34). Moreover, in this study we did not measure body composition. And as such, we cannot rule out that the non-significant reduction in body weight could mask a beneficial body re-composition (by reducing fat mass and increasing lean mass) as a result of healthy lifestyle adaptions.

Furthermore, in the present study, participants in the IG were encouraged to evaluate their hunger and fullness feelings around meals in the DTx. Many individuals with obesity report no clear connection between their eating behavior and sensations of hunger and fullness—a pattern linked to higher disinhibition and hunger scores on the TFEQ (35). Disinhibition refers to the tendency to overeat in response to various stimuli (36). Individuals with high disinhibition scores also tend to prefer high-fat, palatable foods and show a weak satiety response to those foods (37, 38). Higher scores are also linked to susceptibility of weight gain over time and is seen as a predictor of both quantity and quality of food intake (39). In the present study, the IG showed a significant decrease in disinhibition scores (as measured by the TFEQ) during the 12-week intervention warrants further investigation. For example, weight loss induced by a very-low energy-diet alone was previously shown to have little or no effect on these eating behavior traits (40), and the lack of improvements was accompanied by weight regain at the 1-year mark. In contrast, initial decreases in disinhibition scores have been shown to predict WL at 12 months (41).

Another factor with implications for BMI or weight gain is emotional eating (42). Emotional eating is the urge to eat in response to both positive and negative emotions. Recent studies indicate that over 50% of individuals seeking obesity treatment are experiencing emotional eating (43), and it is more common in females (44). On the other hand, self-esteem (treating oneself kindly in times of increased distress or difficulty) (45) has the potential to support both a healthier lifestyle and enhance weight management outcomes (46). Even though the current sample size was small, the results showed an overall trend for reduction in emotional eating. Simultaneously, both groups also experienced significant increases in self-esteem, whereas the IG also showed significant increases in the overall IWQOL-lite score, indicating significant improvements in mental well-being and self-care.

Our overall findings align with prior studies (12) showing that after 3.5 months of participation in Healthy Lives Centers, individuals with- or at risk of developing non-communicable diseases, increased physical activity levels, improved self-reported health, and enhanced health-related quality of life. Yet, the availability of Healthy Lives Center services in Norway *today* varies significantly, and are subject to financial constraints, thus making its potential impact on public health highly vulnerable and inaccessible. Importantly, our results also align with studies showing that digital interventions focusing on self-monitoring, psychological support, and satiety perception have demonstrated reductions in maladaptive eating behaviors and improvements in quality of life (15, 47). Together, these findings support the notion that targeting eating behavior traits and well-being directly through digital tools—rather than focusing solely on weight outcomes may contribute to long-term success in obesity care and help prevent weight regain (41).

The present study only investigated short-term effects, and as such we cannot draw conclusions on the sustainability of these changes. Nevertheless, the observed improvements in central adiposity, eating behavior traits, and quality of life suggest that digital therapeutics may have potential as supportive tools in obesity care. Importantly, our results highlight the need to move beyond short-term changes in body weight and adopt a holistic approach that emphasizes overall health improvements—factors that are critical for long-term success. This perspective aligns with the broader view that obesity should not be defined solely by body weight or BMI, but recognized as a complex disease with metabolic, functional, and psychological dimensions (17). Within this framework, mHealth solutions may play a pivotal role to deliver accessible obesity management and prevention (47).

The greatest strength of this study is its novelty by addressing outcomes beyond weight loss for digital obesity care and -prevention strategies in a municipal healthcare setting. This allows for a timely and urgent evaluation of the efficacy and feasibility of such interventions—a critical issue that has not yet been addressed. Furthermore, it is a strength that this study was in a real-life, clinical setting in the municipality. Importantly, the feasibility of integrating a digital therapeutic into existing municipal Healthy Life Centres indicates that such solutions could be scalable and accessible within primary care systems. The study also has some limitations. Firstly, due to the nature and time constraints of the project, the sample size was small and with a short intervention period. Moreover, attrition is a critical factor in obesity care, and were larger than expected in the present study. Most dropouts occurred around the 12-week follow-up assessments, which coincided with the holiday period. Based on feedback from participants and HLC staff, the main reasons for attrition were lack of time and scheduling difficulties rather than dissatisfaction with the intervention. Importantly, no participant explicitly withdrew due to adverse effects of the digital therapeutic or standard care. Although the current trial was limited by small sample size, attrition, and lack of an intention-to-treat analysis, the large effect sizes observed for several outcomes provide a rationale for future trials.

## Conclusions

The DTx intervention, delivered as an add-on to usual care in a municipal primary healthcare setting, was associated with reductions in central obesity, improvements in reward-related eating behaviors, and enhancements in self-esteem and overall quality of life among individuals with overweight and obesity. These preliminary findings suggest that digital therapeutics may represent a feasible and scalable strategy to support behavioral change and psychological well-being in community-based obesity care. However, given the pilot design and short follow-up, the results should be interpreted as exploratory. Larger, adequately powered, and longer-term trials are needed to establish clinical effectiveness, sustainability of effects, and potential impact at both individual and societal levels.

## Data Availability

The datasets presented in this article are not readily available because we do not have approval to share the data. Requests to access the datasets should be directed to marthe.i.aukan@ntnu.no.
