# Supplementary material for: Beyond weight loss: digital therapeutic for behavioral change and psychological well-being for individuals with overweight and obesity in a primary healthcare setting—A randomized controlled pilot study
Source: Front Digit Health. 2025 Sep 16;7:1671649. doi: 10.3389/fdgth.2025.1671649 (PMC12479526; doi:10.3389/fdgth.2025.1671649)

# Supplementary materials

## **Beyond weight loss: Digital support for behavioral change and psychological well-being for individuals with overweight and obesity in a primary healthcare setting - A randomized controlled pilot study.**

#### **Authors**: Marthe Isaksen Aukan^1,2,^, Maria Arlèn Larsen^3^, Tone Iren Melan^4^, Øyvind Olav Salvesen^5^

#### ^1^ Exercise, Cardiometabolic Health and Reproduction (EXCAR) Research Group, Department of Circulation and Medical Imaging, Norwegian University of Science and Technology (NTNU), Trondheim, Norway; ^2^ Obesity Research Group, Department of Clinical and Molecular Medicine, Faculty of Medicine, Norwegian University of Science and Technology (NTNU), Trondheim, Norway; ^3^Department of Clinical Medicine, UiT The Arctic University of Norway, Tromsø, Norway; ^4^Healthy Lifes Center, Stjørdal Municipally, Stjørdal, Norway; ^5^Department of Public Health and Nursing, Faculty of Medicine, Norwegian University of Science and Technology (NTNU).

##### **Correspondence and reprint requests:** Marthe Isaksen Aukan, Department of Circulation and Medical Imaging, NTNU, Hjerte- lunge senteret (AHL), Prinsesse Kristinas gate 3, 7030 Trondheim, Norway. E-mail: [marthe.i.aukan@ntnu.no](mailto:marthe.i.aukan@ntnu.no)


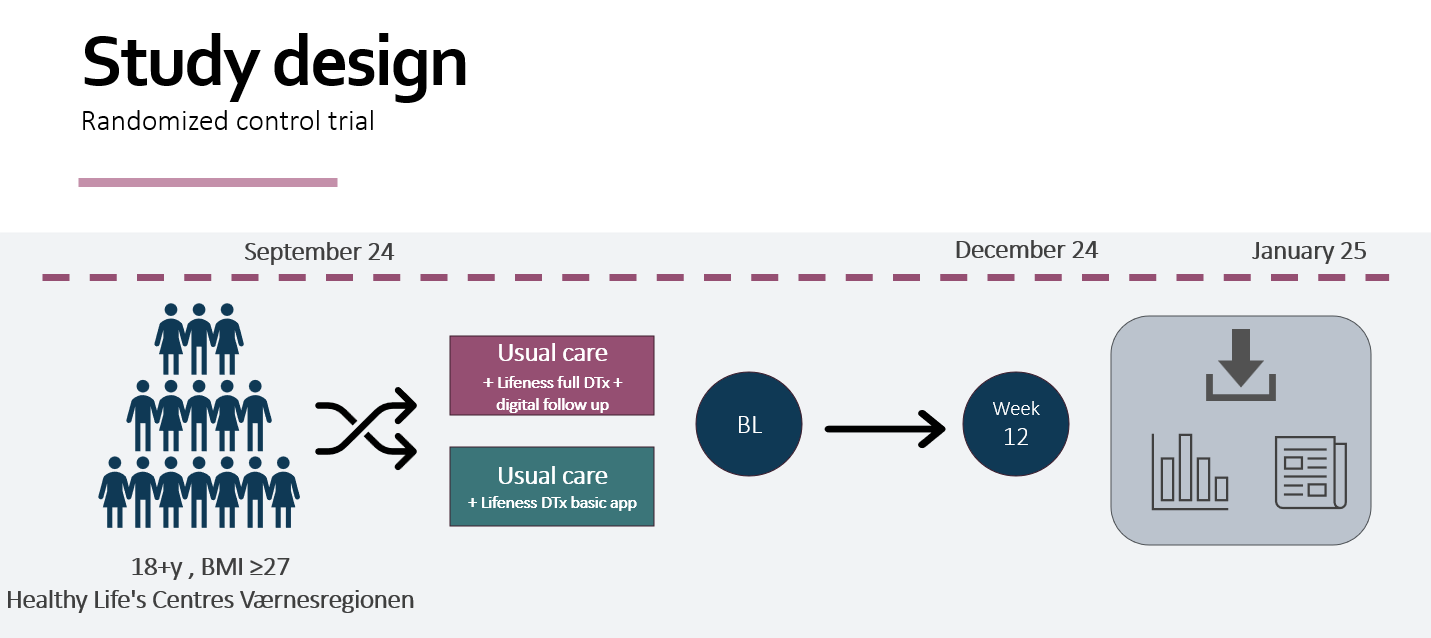


Figure S1. Study design


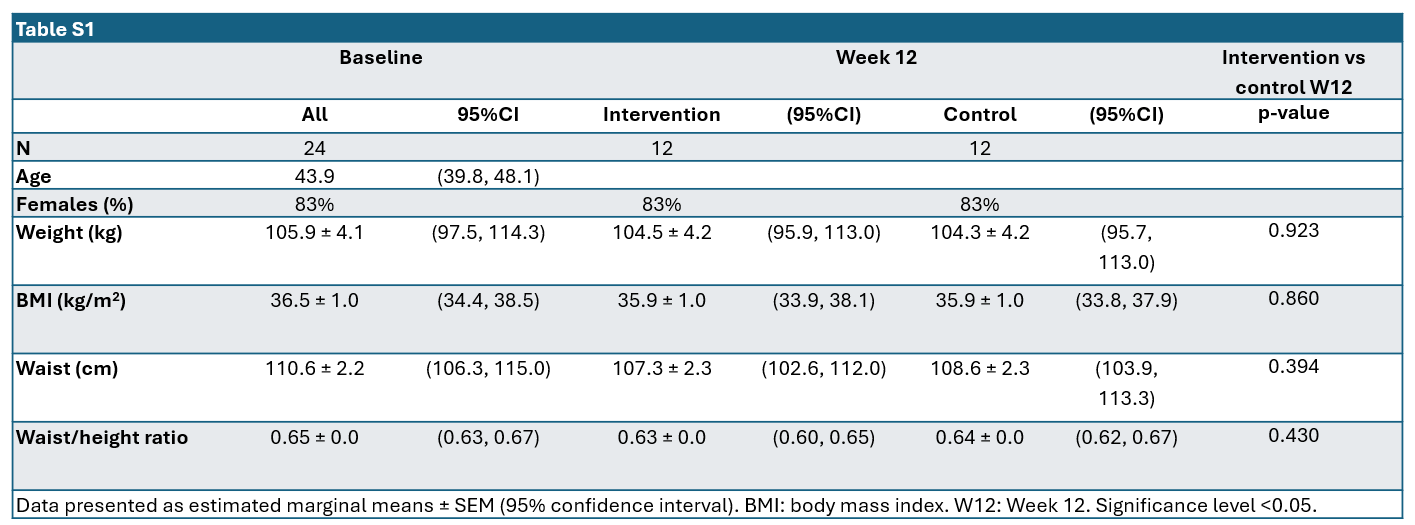


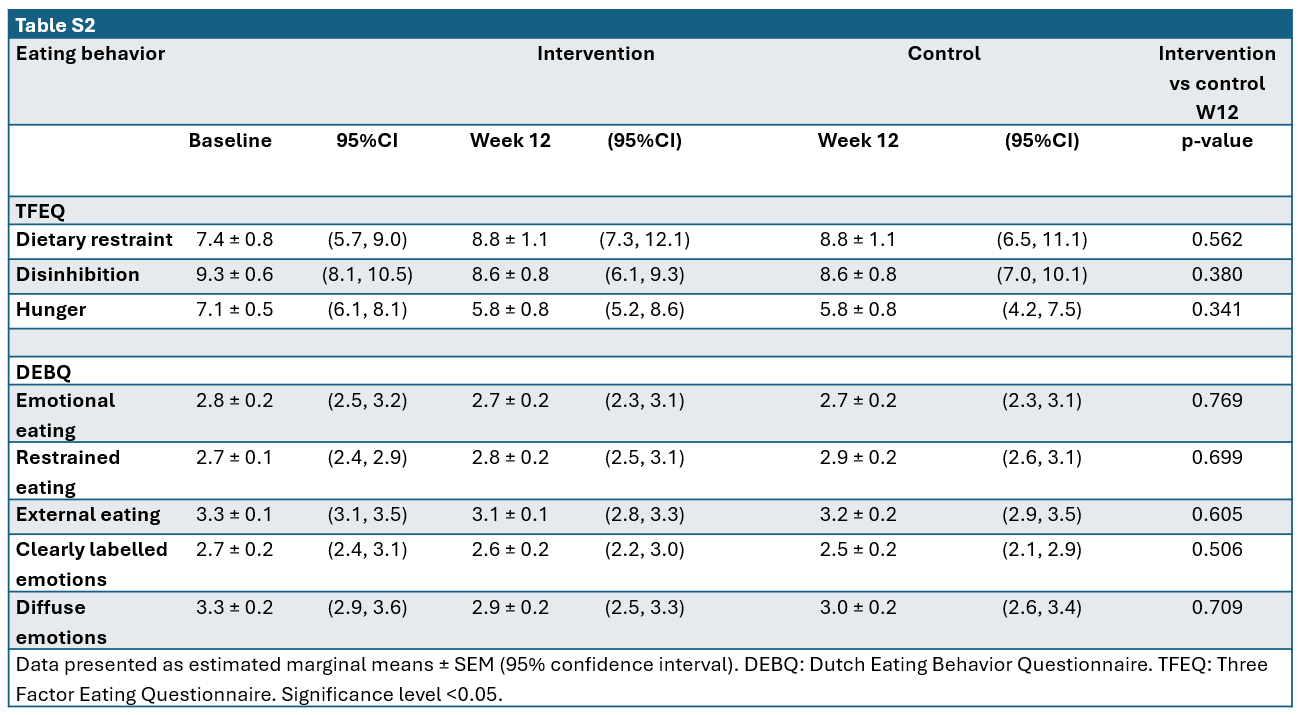


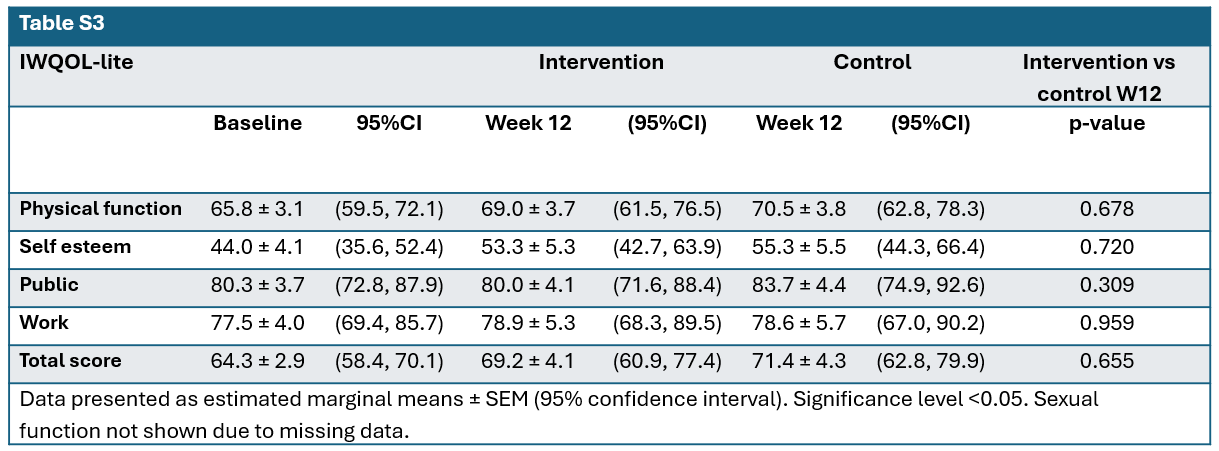

Supplement: Supplementary file 1 [file Datasheet1.docx]
